# Supplementary material for: When Matilda shows up: The double-edged impact of women researchers’ media visibility in management science
Source: PLoS One. 2026 May 27;21(5):e0348097. doi: 10.1371/journal.pone.0348097 (PMC13215518; doi:10.1371/journal.pone.0348097)
Supplement: Attachment — Submitted filename: Plos One letter to reviewer 11 AUGUST - BP.docx [file pone.0348097.s003.docx]

**When Matilda shows up:**

**The double-edged impact of women researchers’ media visibility**

***(Manuscript Number: PONE-D-25-25301)***

**General comment**

Dear Editors,

Dear Reviewers,

We are grateful for your thoughtful and detailed feedback on our manuscript. We have revised the manuscript extensively in response to your comments and suggestions. Below, we provide a detailed, point-by-point response. Each reviewer’s comment is reproduced in full, followed by our reply and a description of the changes made to the manuscript.

*	*

*

**Detailed responses to Reviewer 1**

**Abstract**

**The first sentence uses ‘women,’ which indicates gender and ‘female’ which indicates sex. Please investigate how these terms are different and consider which is more appropriate for this study. I think that using terms that reflect gender is most important. People who appear as women have an impact on others who also identify as women.**

**Reply:** We agree and have revised all uses of “females” and “males” to “women” and “men” in the abstract and throughout the manuscript where gender (not sex) is the relevant concept.

**It is unclear whether the gains (increased perceived expertise and decrease the gender gap for self-efficacy beliefs) is specific to the public, grads, or both. Please include null results in the abstract as well.**

**Reply:** We clarified in the abstract which effects apply to which populations and have added a sentence to acknowledge the non-significant findings. This is how it now appears:

*Although the proportion of women in academic positions has increased, women researchers continue to receive less media coverage than their counterparts. To examine the implications of this underrepresentation, we investigate how non-academic audiences respond to women researchers’ increased visibility in science communication media. We conducted two laboratory experiments, the first with a sample of non-academic individuals (N = 271) and the second with a sample of graduate students (N = 129).* ***Results show that greater visibility of women researchers reduces the gender gap in self-efficacy beliefs between men and women in both populations. It also increases women researchers’ perceived expertise among non-academic participants, but not among graduate students. Among the later, however, heightened visibility is also unexpectedly associated with a decline in the perceived attractiveness of academic careers, but only for men.*** *While our research focuses specifically on the field of management and measures media visibility through the relative representation of women and men in a simulated newsletter, our findings provide broader insights into how media exposure can shape self-efficacy beliefs, researchers’ perceived expertise, and the appeal of academic careers. Building on these insights, we propose recommendations for researchers, academic institutions, and media organizations involved in science dissemination.*

**Temper language to ensure that “management science” is not being generalized to “science” and that a “newsletter” is not being generalized to all “media.”**

**Reply:** Language has been revised in the abstract and throughout the manuscript to reflect the specific disciplinary (management science) and medium-specific (newsletter) nature of our study. This is how it now appears in the abstract:

*Although the proportion of women in academic positions has increased, women researchers continue to receive less media coverage than their counterparts. To examine the implications of this underrepresentation, we investigate how non-academic audiences respond to women researchers’ increased visibility in science communication media. We conducted two laboratory experiments, the first with a sample of non-academic individuals (N = 271) and the second with a sample of graduate students (N = 129). Results show that greater visibility of women researchers reduces the gender gap in self-efficacy beliefs between men and women in both populations. It also increases women researchers’ perceived expertise among non-academic participants, but not among graduate students. Among the later, however, heightened visibility is also unexpectedly associated with a decline in the perceived attractiveness of academic careers, but only for men.* ***While our research focuses specifically on the field of management and measures media visibility through the relative representation of women and men in a simulated newsletter, our findings provide broader insights into how media exposure can shape self-efficacy beliefs,*** ***researchers’ perceived expertise, and the appeal of academic careers.*** *Building on these insights, we propose recommendations for researchers, academic institutions, and media organizations involved in science dissemination.*

**Introduction**

**In section 1 it seems odd that the impact on an academic audience (graduate students) is not mentioned here given the sample focused on that population.**

**Reply:** We have updated the introduction in several ways following your comment. First, we acknowledge that graduate students could be considered an academic audience and have changed the title of the article from “When Matilda shows up: The double-edged impact of women researchers’ media visibility on a non-academic audience” to a more neutral “When Matilda shows up: The double-edged impact of women researchers’ media visibility”.

Second, your feedback made us realize that we had not sufficiently addressed the question of the target audiences for scientific dissemination activities in the introduction and the rationale for focusing on the general public and on graduate students in our research. We have therefore clarified these aspects as follows:

*Despite the general rise in academic visibility, research dissemination in newspapers, radio and television, news websites, and dedicated online platforms seems to make little room for women researchers. Men also communicate more than women on social media [9] and personal blogs [13]. Women have made up some ground recently, but only 24% of expert voices heard in the news in 2020 were female (vs. 19% in 2015 ). Women researchers’ underrepresentation in the media is considered an enduring trend [4] with significant negative consequences. The extant research shows that this underrepresentation is detrimental to women academics’ careers [14,15]* ***but has not explored its impact beyond peers from the same discipline and academic institutions. Yet scientific dissemination efforts are increasingly directed toward broader lay audiences [6,9], including the general public, to foster scientific literacy and engagement, the educational community, to integrate research findings into teaching and training and decision-makers and socio-economic stakeholders, to inform policies and professional practices. In this article, we specifically examine how the media visibility of women researchers influences two key audiences: the general public, as the primary target of scientific dissemination activities, and graduate students, who may be closer to the academic realm but for whom role models are particularly important in envisioning an academic career****.*

Third, following your next comment, we removed the summary of our findings from the end of the introduction and instead expanded the description of our empirical approach to clarify that we conducted two distinct experiments: one with a sample from the general public and the other with a sample of graduate students. This section now reads as follows:

*In this article, we explore whether the visibility of women researchers in scientific dissemination media could enhance audience’s perceptions of women researchers’ expertise, self-efficacy beliefs, and the attractiveness of an academic career.* ***To address this question, we conducted two laboratory experiments: the first with a sample of non-academic individuals (N = 271), representing the primary target of scientific dissemination activities, and the second with a sample of graduate students (N = 129), who may be closer to the academic sphere but for whom role models are particularly important in envisioning an academic career.***

**The introduction should be framed with this experiment in mind. Currently, these experiments are largely absent from the intro.**

**Reply:** As you can see above, we have added further details to present the two experiments from the outset in the introduction.

**End of section 1: It seems a bit unusual to summarize the findings here.**

**Reply:** We have removed the summary from the end of Section 1 and repositioned it more appropriately in the Discussion section.

**Section 2**

**Section 2.2. The word “refusing” seems strong here and perhaps an overstatement. “Decline” may be more appropriate.**

**Reply:** We have replaced “refusing” with “declining,” as suggested.

**Section 2.3.1 The sentence that says that lower media visibility weakens careers, salaries, self-esteem and wellbeing is a bit too strong. I suggest rephrasing by adding “can” weaken.**

**Reply:** We have softened this claim by stating that lower media visibility “can weaken” these outcomes.

**Section 2.3.3: It is unclear what is meant by counter-stereotypical women in research. This implies that the woman herself is counterstreotypic.**

**Reply:** We have rephrased to refer to “counter-stereotypical portrayals of women,” making clear that we refer to representation, not individual identity.

**Section 2.3.3: Paragraph after H1 (pg 10) sentence in the middle of a paragraph abruptly cuts off.**

**Reply:** We have identified and corrected the incomplete sentence.

**Section 3**

**Section 3.1**

**What is management science? Is this considered STEM? As a reviewer in the US, I was unfamiliar with this field and think many readers would appreciate a brief explanation and also what distinguishes it as a STEM field.**

**Reply:** Thank you for raising this point. To avoid confusion and better reflect the scope of our stimuli, we have replaced the term “management science” with “management” throughout the manuscript. This broader term aligns more closely with the range of topics included in the newsletter stimuli and avoids suggesting a strict classification as a STEM field.

**Typo near citation 121**

**Reply:** Corrected.

**How were graduate students recruited specifically? What language was used in the recruitment for the study? How were participants incentivized?**

**Reply:** We expanded the Method section to include the missing information as follows:

*We recruited 148 business school graduate students to participate in Study 2.* ***Participants were contacted through HEC Montréal’s graduate student mailing list, completed the survey voluntarily without any incentive, and responded in French.*** *Study 2 followed the same protocol as Study 1, with two notable exceptions.*

For the sake of symmetry, we specified for Study 1 that the sample was composed of French participants:

*We recruited 347* ***French*** *participants through the online panel of a European professional market research institute.*

**In what ways were the participants told to interact with this newsletter? Presumably no lay person is going to randomly interact with a newsletter about management science. As such, how do you view this as an exercise that reflects lay people’s interaction with the media more broadly?**

**Reply:** We now explain that participants were instructed to read the newsletter content carefully as part of the experimental design. We also clarify that our intervention simulates structured rather than organic exposure. In addition, we specify the online platforms in France that inspired our design, one of which has over 116k followers on YouTube. This indicates that lay audiences are indeed interested in engaging with online research dissemination newsletters that they can subscribe to in order to receive the latest updates.

*The design of our experimental stimuli was inspired by actual newsletters distributed by online platforms that aim to popularize academic research* ***(e.g., Xerfi Canal on YouTube, which displays more than 116k followers as of August 12, 2025).*** *To maintain ecological validity, the men-dominated condition, featuring eight men researchers out of nine, mirrors real patterns of research dissemination observed in one of the authors’ home countries […]*

***To simulate deliberate rather than incidental media exposure, we explicitly instructed participants to read the newsletter content carefully, as they would be asked questions about its content afterward. We acknowledge that this structured exposure differs from organic media consumption; however, it is common and necessary in experimental settings where stimulus control is essential.*** *After exposing participants to our stimuli, we measured their general self-efficacy beliefs. We then showed them a presentation of a research project being undertaken by “Clémence Dubois”, a fictitious woman researcher. It contained a short description of her fictitious project and a picture generated by the same AI used for the initial stimuli (see Figure 3).*

**The paper should acknowledge the presence of gender nonbinary and gender queer individuals. The paper can acknowledge their existence in science while still** **focusing on gender binary categories. Relatedly, were participants given the option to identify as gender non-binary?**

**Reply:** We now detail in the Method section how gender was measured. We also report the number of participants who did not identify as a man or a woman and explain that they were excluded from the analyses due to the very small subgroup size: 3 participants in Study 1 (2 who described themselves as “human beings” and 1 who did not provide an answer) and 3 participants in Study 2 (1 who self-identified as “gay” and 2 as “non-binary”). This is how it now appears for Study 1:

*Towards the end of the survey, participants were asked to indicate their gender (“woman,” “man,” “non-binary,” “prefer to self-describe [free-text field] ,” or “prefer not to say”), age, and highest educational qualification. […] We excluded from the analyses 3 participants who did not identify as either a man or a woman in order to focus on gender binary categories and 73 participants who provided an incorrect response regarding the gender composition of the newsletter.*

**I strongly disagree that “To what extent do you feel confident in your ability to express your opinion on a management issue” is reflective of perceived attractiveness of an academic career. Is this a previously validated scale to measure attractiveness of an academic career? I think this is a typo after looking at Table 1—this is a self-efficacy question.**

**Reply:** You are absolutely right. This was a mistake. This item was a self-efficacy item indeed. We corrected it to properly show a perceived attractiveness of an academic career item : “*To what extent do you feel academia would be a good work environment for you?*”

**Are business school graduates studying “management science”?**

**Reply:** We replaced management science with management throughout the manuscript.

**The claim that low media visibility of women makes academic carers less attractive to them than for men is incorrect because the experiment would suggest the attractiveness of a career for women is not dependent on representation (but it is for men).**

**Reply:** You are right. We have modified our claim accordingly:

*Specifically, women researchers’ lower media visibility has two critical impacts.* ***It can weaken young women’s self-efficacy beliefs before they have even embarked on an academic career and may lead them to feel less legitimate in aspiring to such a career.*** *It may then limit women researchers’ advancement once they have embarked on an academic career, by driving a perception that they are generally less expert than their counterparts.*

**I’m not sure it’s correct to say that other-gender role models can be counterproductive for men. Perhaps men have inflated self-efficacy and this helps calibrate it?**

**Reply:** We have eliminated this idea from the discussion.

**Section 5**

**Section 5.1. A newsletter should not be extrapolated to represent “media.” As such many claims in the discussion are too strong such as, “confirming that media’s representation of the world has negative performance effects not just on academia but on society in general.”**

**Reply:** Thank you for pointing this out. We have revised Section 5.1 to more accurately describe the scope of our findings. We now clarify that our experimental stimuli represent a specific and structured form of media exposure, namely, a newsletter highlighting research contributions. Accordingly, we have tempered general claims about “the media” and now frame our conclusions in terms of the potential effects of gendered academic representation in public-facing communication, rather than drawing broad conclusions about all media.

**Please temper all language in the discussion and do not generalize “management science” to science or STEM. Similarly, specify “newsletters” as opposed to “media.”**

**Reply:** We have now made consistent use of “management” across the manuscript, and specify in the discussion that our results don’t apply to all researchers, but specifically to management scholars. As an addition, we have added avenues for research in the very last section of our manuscript:

*The experimental design could also be extended to other forms of media, such as podcasts, television programs, or popular science magazines, in order to test whether the observed effects hold across different dissemination channels. Likewise, applying a similar approach to other academic disciplines beyond management, such as natural sciences, engineering, medicine, or the arts, could reveal whether the dynamics we observe are discipline-specific or more generalizable.*

**Section 5.3. The first paragraph of the conclusion should be labeled limitations.**

**Reply:** We labeled and expanded this paragraph as a “Limitations and further research” section, as suggested.

*	*

*

**Reviewer #2**

**Thank you for the opportunity to review this work, I found it very interesting and worthwhile. This manuscript addresses a timely and socially significant issue: the impact of female researchers’ media visibility on public perceptions and academic career aspirations. The authors employ two well-structured experimental studies to explore how gender representation in media influences perceived expertise, self-efficacy beliefs, and the attractiveness of academic careers. The paper is clearly written, well-organized, and grounded in relevant theoretical frameworks, including social role theory.**

**Reply:** We sincerely thank the reviewer for their encouraging assessment and insightful suggestions. Please find our responses to each specific comment below.

**The introduction is particularly comprehensive, offering a detailed review of the literature that effectively situates the study within broader academic and societal debates. However, there is an over-reliance on direct citations, which detracts from the authors’ own voice. Rephrasing some of these references in the authors’ own words would help clarify how each cited work supports the study’s rationale and hypotheses, and would better integrate the literature into the narrative.**

**Reply:** We revised the introduction to reduce excessive use of direct citations, integrating references more seamlessly into our own narrative voice. However, we retained two quotes (i.e., one from Oscar Wilde in the opening sentence and one from the Nobel acceptance speech of Emmanuelle Charpentier) as we believe these lend rhetorical impact and effectively frame the broader societal relevance of our study.

**Additionally, the manuscript currently uses a mix of numerical and in-text citation styles, which should be standardized for consistency and clarity.**

**Reply:** We have fixed that

**While the experimental design is methodologically sound and I appreciated the rationale behind the choice of design and stimuli, the manuscript would benefit from more cautious use of causal language. Phrases suggesting that media visibility “increases” self-efficacy or “erodes” perceptions may overstate the findings, particularly given the complexity of the social and psychological processes involved. A more measured phrasing—such as “is associated with” or “may influence”—would better reflect the limitations of experimental inference in this context.**

**Reply:** We have carefully revised all such statements to reflect more cautious, correlational phrasing.

**Similarly, the formulation of Hypothesis 1 could be improved for clarity. The term “increase” is somewhat vague and would benefit from a more precise definition. It is not immediately clear whether the authors are referring to more positive perceptions, higher ratings on specific scales, or another outcome. As such, I recommend explicitly linking the hypothesis to the operationalized measures.**

**Reply:** We revised Hypothesis 1 to be more precise. Here is how it now appears:

***H1****:* *Greater women researchers’ visibility in scientific dissemination media will lead to higher perceived expertise of women researchers compared to lower women’s visibility.*

We have rewritten the two other hypotheses for the sake of consistency.

**In a similar vein, the conclusions drawn in the discussion section should be more carefully quantified. While the study provides valuable insights into perceptions and self-reported beliefs, it does not measure actual career advancement or long-term outcomes, which the text sometimes alludes to. As such, claims about the broader impact on academic career trajectories should be moderated to reflect the scope of the data. Acknowledging this limitation more explicitly would strengthen the credibility of the conclusions.**

**Reply:** We tempered these claims and clearly acknowledged the short-term and perceptual scope of our data. This is how it now appears:

*Once they have embarked on an academic career, awareness that they may be perceived as less expert than their counterparts because of their lower visibility could undermine their confidence and potentially hinder their long-term advancement.*

**Minor issues:**

**There are also some minor issues with redundancy and structure. For instance, the phrase “between-subjects laboratory experiments” is repeated unnecessarily on page 12 and could be streamlined.**

**Reply:** We removed the redundant phrasing.

**Furthermore, while the results of Study 1 are clearly presented, the manuscript would benefit from a brief discussion section following this study. Summarizing the key findings and their implications before transitioning to Study 2 would help maintain narrative coherence and reinforce the significance of the results.**

**Reply:** We added a brief transition paragraph summarizing Study 1 before introducing Study 2.

*Study 1’s results suggest greater visibility of women researchers in scientific dissemination media can enhance their perceived expertise and reduce gender gaps in self-efficacy beliefs, highlighting the potential of such media to shape public perceptions. Study 2 aims to assess the robustness of these findings by examining whether individuals who are more familiar with the academic world, namely graduate students, are less susceptible to biases stemming from women researchers’ invisibility. Its second objective is to test H3, which could not be examined in Study 1, as surveying individuals from the non-academic general population would not allow for measuring a genuine intention to pursue an academic career.*

**In terms of statistical reporting, the manuscript currently reports p-values in threshold form (e.g., p < .05). Providing full p-values would enhance transparency and allow readers to better assess the strength of the evidence. Given the nuanced nature of the findings and the fact that one of the effects was not replicated across the 2 studies, I also encourage the authors to add effect sizes to their results.**

**Reply:** In the new version of the manuscript, we provide full p-values and partial eta squared for all significant results.

*	*

*

We hope that our revisions adequately address your suggestions and improve the clarity and rigor of our manuscript. We thank you again for your time and valuable insights.
