# Supplementary material for: When Matilda shows up: The double-edged impact of women researchers’ media visibility in management science
Source: PLoS One. 2026 May 27;21(5):e0348097. doi: 10.1371/journal.pone.0348097 (PMC13215518; doi:10.1371/journal.pone.0348097)
Supplement: Attachment — Submitted filename: PLOS1 - Response to reviewers - R2 Final.docx [file pone.0348097.s004.docx]

Before addressing each comment in detail, we would like to express our sincere appreciation to both reviewers for their thoughtful and constructive feedback. We are particularly grateful to Reviewer 2, who indicated that our responses sufficiently addressed heir concerns and that the manuscript could be accepted in its current form. We carefully considered all remaining suggestions from Reviewer 1 and revised the manuscript accordingly, as detailed below.

**Reviewer #1 Comment 1 :  Management is not a natural science and yet much of the introduction draws from literature specific to the underrepresentation of women in science, STEM, and science communication (which often exclusively is specific to the natural sciences). Management is treated by the European Research Council within Social Sciences & Humanities. Similarly, the OECD/UNESCO FOS system classifies business and management as a social science. As such, the paper needs to be fully revised to reflect this important classification.**

Before addressing Reviewer 1’s comments point by point, we would like to note that fully restructuring the manuscript is challenging at this stage, particularly as Reviewer 2 indicated that the paper could be accepted in its current form. Nonetheless, we carefully reflected on Reviewer 1’s suggestions and made substantial revisions across multiple sections of the manuscript. Specifically:

- We changed the title to explicitly specify the disciplinary scope by adding “management science”;
- We modified the introduction to more clearly establish the cross-disciplinary relevance of the phenomenon and to justify our empirical focus on the social sciences, particularly management science;
- We extended the literature review to highlight that many studies reviewed in Table 1 (marked with an asterisk) draw on social science data, showing that research on women’s lower visibility is not limited to STEM fields but also occurs in disciplines perceived as more gender-balanced, thereby reinforcing the cross-domain relevance of gendered visibility dynamics;
- We now explicitly specified “social science dissemination media” at key anchor points;
- We extended the discussion section, to include a limitation regarding the fact that the experiments only considered the field of management science.

These changes are described in greater detail in our responses below.

**Reviewer #1 Comment 4 :  Further, the introduction should be revised to focus only the representation of women in the social sciences or preferably business/management research and exclude STEM and the natural sciences. Many of the references the authors make, including many of the papers cited in Table 1, are specific to STEM or the natural sciences, which is extremely misleading for the reader.**

We appreciate your emphasis on positioning management within the social sciences, and we fully agree with this classification. We have clarified this explicitly in the introduction.

At the same time, we would like to emphasize that the literature on gender bias in researchers’ visibility has developed largely in a cross-disciplinary manner rather than being confined to specific scientific domains. Among the 42 articles summarized in Table 1, approximately 45% explicitly include the social sciences, either as a primary focus or within a cross-disciplinary approach, while the remaining studies focus on STEM or life sciences. This reflects the prevailing approach in this research stream, in which shared mechanisms, such as gender stereotypes, role modeling, cumulative advantage, and institutional bias, are theorized as operating across academic fields rather than being domain specific.

Importantly, these theoretical frameworks are not rooted in the natural sciences but are widely applied in social science research on inequality and organizations. There is therefore little reason, based on the existing literature, to assume that mechanisms identified in one scientific domain would not operate in another.

Moreover, from a substantive perspective, examining these dynamics within management constitutes a contribution rather than a limitation. Gender bias is often presumed to be more salient in STEM and life sciences (e.g., Leslie et al., 2015; Banchefsky & Park, 2018). Demonstrating that similar processes shape media visibility in management and the social sciences, where gender-science stereotypes are commonly assumed to be weaker, thus provides particularly informative insights.

We thank you for encouraging us to clarify these points, which helped us improve the precision of the manuscript. Accordingly, we made the following changes.

First, we revised the introduction to position prior work as cross-disciplinary and to clarify that the present study extends these insights specifically to the field of management. We also added a dedicated paragraph to justify the relevance of management as the focal discipline and to articulate its ecological validity for the present research. This paragraph now reads as follows:

*Building on these perspectives, we posit that women researchers’ lower visibility in scientific dissemination activities has the potential to reshape social representations of both research and researchers. In the present work, we investigate this phenomenon in the social sciences, focusing specifically on management science. We do so for two reasons. First, management science increasingly engages in research dissemination aimed at broad audiences and plays a central role in shaping organizational practices, leadership norms, and public discourse, making visibility dynamics in this field particularly consequential. Second, studying these dynamics in management science provides a conservative test: gender-science stereotypes are typically presumed to be weaker in the social sciences than in STEM and life sciences (e.g., Leslie et al, 2015; Banchefsky & Park, 2018). Demonstrating comparable visibility effects in this context therefore offers especially compelling evidence of the pervasiveness of gendered evaluative processes. Accordingly, we argue that these effects are likely to extend across all academic domains, from STEM to the social sciences.*

Second, we now indicate this in Table 1 by marking with an asterisk the studies that draw on social science data, either as a primary focus or within a cross-disciplinary approach, thereby clarifying that our literature review builds on prior research spanning multiple academic domains. We also added the following paragraph:

*Importantly, a substantial number of the studies reviewed in Table 1 (i.e., references marked with an asterisk), draw on data from the social sciences. This suggests that lower visibility is not confined to STEM or life sciences, where gender bias is often assumed to be most pronounced, but is also documented in disciplines typically perceived as more gender-balanced. The persistence of these patterns in the social sciences reinforces the argument that gendered visibility dynamics reflect broader evaluative processes operating across academic domains.*

Third, we extended the discussion section, to include a limitation regarding the fact that the experiments only considered the field of management science.

*A further limitation is that we examined these dynamics exclusively within the field of management. Although this focus limits generalizability, it also constitutes a substantive contribution, as gender bias is often presumed to be more salient in STEM and life sciences. Showing that similar dynamics operate in management and the social sciences—where such stereotypes are assumed to be weaker—offers particularly informative insights. The experimental design could therefore be extended to other forms of media, such as podcasts, television programs, or popular science magazines, in order to test whether the observed effects hold across different dissemination channels. Likewise, applying a similar approach to other academic disciplines beyond management, such as natural sciences, engineering, medicine, or the arts, could reveal whether the dynamics we observe are discipline-specific or more generalizable.*

**Reviewer #1 Comment 2 :  The title should specify “women researchers’ media visibility in the field of management.”**

Thank you for this helpful suggestion. We agree that explicitly specifying the disciplinary context strengthens the clarity and positioning of the article. We therefore revised the title to: “When Matilda Shows Up: The Double-Edged Impact of Women Researchers’ Media Visibility in Management Science.” This revised title preserves the conceptual framing while clearly anchoring the study within the field of management, in line with both the experimental stimuli and the theoretical contributions of the paper.

**Reviewer #1 Comment 3 :  The term “scientific dissemination media” should be revised throughout to specify social science dissemination.**

We agree that clarifying the disciplinary scope of the dissemination context strengthens the precision of the manuscript. Accordingly, we now explicitly specify “social science dissemination media” at key anchor points: in the abstract, at the first occurrence in the introduction, and at the first mentions in both the discussion and conclusion. To preserve readability and avoid unnecessary repetition, we subsequently retain the shorter term “scientific dissemination media,” as the social science context is clearly established. This approach ensures both conceptual clarity and stylistic fluidity.

**Reviewer #1 Comment 5 :  The authors continue to conflate the non-academic audience and graduate student audience. The paper needs to be fully revised to reflect that this set of experiments examines the impact on social science researcher media visibility on two different audiences, one of which is non-academic and the other which is actively working within an academic system.**

We took your previous comment from the first round of review into account; however, an unintended reference to a “non-academic audience” remained in the abstract in Revision 1. We agree that this wording required correction and have now revised it accordingly.

**Reviewer #1 Comment 6 :  Please specify what the online panel of European market research institute is.**

We are now mentioning PanelLabs as the online panel of European market research institute.

**Reviewer #1 Comment 7 :  Please provide descriptive demographics (e.g., gender, race/ethnicity, college generation status) of the participants for study I and study II.**

We were already giving age and gender as demographics for both studies. We added level of study for Study I. We unfortunately don’t have race/ethnicity or college generation status. We agree that these could play a role in our study, and are now mentioning this in the limitations and further research.

**Reviewer #1 Comment 8 :  For Study 2 methods, please reiterate that HEC is a business school.**

We had already indicated in the Method section that “we recruited 148 business school graduate students to participate in Study 2. Participants were contacted through HEC Montréal’s graduate student mailing list.” However, we acknowledge that this may not have been sufficiently explicit. We therefore revised this passage to: “We recruited 148 graduate students enrolled in a business school (HEC Montréal) to participate in Study 2. Participants were contacted through the school’s graduate student mailing list, completed the survey voluntarily without any incentive, and responded in French.” We hope this revised wording provides greater clarity.

**Reviewer #1 Comment 9 :  Please be more specific in your language (e.g., page 18- suggesting weaker gender-science stereotypes among people who are more familiar with the academic world). First, the reader does not know what gender stereotypes exist in management research specifically. And, I think the more important difference between the non-academic audience and the graduate student audience may be that the grad audience is familiar with the “academic world of management” and not the “academic world” broadly.**

We fully agree that this sentence in the Method section was misleading. Nonetheless, we believe it is noteworthy that in Study 1, 73 participants failed the manipulation check (i.e., recalling whether they had seen men or women in the newsletter), whereas in Study 2 only one graduate student did so. We therefore added a brief footnote to acknowledge this difference, without speculating on its underlying causes.

In addition, we revised the manuscript throughout to clarify the “more familiar” argument, specifying that the graduate student sample is familiar with the academic world of management rather than with academia more broadly. We thank the reviewer for this helpful clarification.
